# Identification of Novel Viruses Using VirusHunter -- an Automated Data Analysis Pipeline

**DOI:** 10.1371/journal.pone.0078470

**Published:** 2013-10-22

**Authors:** Guoyan Zhao, Siddharth Krishnamurthy, Zhengqiu Cai, Vsevolod L. Popov, Amelia P. Travassos da Rosa, Hilda Guzman, Song Cao, Herbert W. Virgin, Robert B. Tesh, David Wang

**Affiliations:** 1 Departments of Pathology & Immunology, Washington University School of Medicine, and the Midwest Regional Center of Excellence for Biodefense and Emerging Infectious Diseases Research, St. Louis, Missouri, United States of America; 2 Department of Molecular Microbiology, Washington University School of Medicine, and the Midwest Regional Center of Excellence for Biodefense and Emerging Infectious Diseases Research, St. Louis, Missouri, United States of America; 3 Department of Pathology, Center for Biodefense and Emerging Infectious Diseases, University of Texas Medical Branch, Galveston, Texas, United States of America; Radboud University Medical Centre, NCMLS, Netherlands

## Abstract

Quick and accurate identification of microbial pathogens is essential for both diagnosis and response to emerging infectious diseases. The advent of next-generation sequencing technology offers an unprecedented platform for rapid sequencing-based identification of novel viruses. We have developed a customized bioinformatics data analysis pipeline, VirusHunter, for the analysis of Roche/454 and other long read Next generation sequencing platform data. To illustrate the utility of VirusHunter, we performed Roche/454 GS FLX titanium sequencing on two unclassified virus isolates from the World Reference Center for Emerging Viruses and Arboviruses (WRCEVA). VirusHunter identified sequences derived from a novel bunyavirus and a novel reovirus in the two samples respectively. Further sequence analysis demonstrated that the viruses were novel members of the *Phlebovirus* and *Orbivirus* genera. Both *Phlebovirus* and *Orbivirus* genera include many economic important viruses or serious human pathogens.

## Introduction

Quick and accurate identification of microbial pathogens is essential for both diagnosis and response to emerging infectious diseases. High-throughput sequencing has recently emerged as a powerful approach to identify both known and novel viruses in clinical specimens. For example, high throughput Sanger sequencing combined with bioinformatic analysis lead to the discovery of human bocavirus [1], KI polyomavirus [2] and WU polyomavirus [3]. With the advent of next-generation sequencing (NGS) technology, the increase in sequencing capability has greatly facilitated efforts to identify viruses in clinical specimens. Many novel viruses have been detected in human specimens, such as Merkel polyomavirus [4], astrovirus VA1 [5] and Lujo virus [6]. Currently, the recognition that sequences are derived from a novel virus is dependent primarily upon detecting sequence similarity between a given read and known reference sequences in various databases. Because longer nucleotide sequence reads can be translated into longer amino acid sequences, they provide a higher probability of detecting divergent sequences distantly related to known viruses. In the past few years, the majority of viruses identified by Next Generation sequencing have utilized Roche/454 platform due to the longer read length compared to Illumina or SOLiD platforms. NGS instruments are now available in most research institutions and the dramatic reduction in sequencing costs has made experimental NGS broadly accessible. However the computational analysis required to identify viral sequences within the massive volumes of data generated by NGS is a barrier for many researchers. To address this limitation, we have developed a robust computational pipeline, VirusHunter, for analysis of NGS data that is suitable for detection of both novel and known virus sequences. Beta versions of VirusHunter have been used to identify both known and novel viruses from a wide array of specimen types [5,7–13]. VirusHunter is freely available from http://www.ibridgenetwork.org/wustl/virushunter .

The World Reference Center for Emerging Viruses and Arboviruses (WRCEVA) maintains a large collection of virus isolates and provides reagents and support for investigations of virus outbreaks throughout the world [14]. This NIAID-funded program also identifies and characterizes arboviruses (arthropod-borne viruses) and other suspected emerging viruses. The repository within the WRCEVA stores over 6000 classified arthropod-borne, rodent-borne, and other zoonotic and human viruses. In addition there are many virus samples in the collection that have not been well characterized or classified. 

Salanga virus (strain AnB 904a) was originally isolated from a rodent (*Aethomys medicatus*) collected in 1971 in Salanga, Central African Republic [15] by researchers at the Institute Pasteur, Bangui, CAR. Initial analyses determined that it was chloroform-sensitive, but it did not react with any of their reagents to known African arboviruses. The virus was then sent to the Institute Pasteur, Dakar, Senegal and subsequently sent to the WRCEVA for further characterization. An early ultrastructural study by El Mekki et al. suggested that Salanga AnB 904a was a Poxvirus [16]. Currently there are two listings of Salanga virus in the International Committee on Taxonomy of Viruses (ICTV) database. One is an unassigned species in the *Bunyaviridae* (acronym SGAV) while the other is an unassigned species in the *Poxviridae* (acronym SGV) [17]. However, since no genome sequence is available; no definitive conclusion can be made about the taxonomic classification of Salanga virus and whether there may be two distinct viruses that currently share the same name. 

KY-663 virus was originally isolated on July 18^th^, 1965 from the blood of a bat (*Myotis macrodactylus*) collected in a mine at Heramatsu, Kagoshima, Japan. The initial isolation was made by intracerebral inoculation of newborn mice. KY-663 virus was found to be partially resistant to treatment with sodium deoxycholate and did not produce a hemagglutinin. Intracranial inoculation of KY-663 into suckling mice causes lethality in 4-5 days, but it is not pathogenic to adult mice [18]. After efforts to identify the virus in Japan were unsuccessful, it was sent to the WRCEVA for further study, but the virus remained unclassified. 

In order to identify and taxonomically classify these two viruses, we used NGS technology to obtain the genomic sequences of those viruses and our customized bioinformatics pipeline, VirusHunter, to analyze the sequences. Furthermore, traditional virologic methods, including electron microscopy and serology, were also employed to provide additional modes of characterization of these viruses. Based on these analyses, Salanga virus was classified as a novel member of the *Phlebovirus* genus in the *Bunyaviridae* while KY-663 was determined to be a novel member of the *Orbivirus* genus in the *Reoviridae*. We tentatively named KY-663 Heramatsu virus (HERMV) in this manuscript.

## Results

### Ultrastructural Characteristics

**Figure 1:**
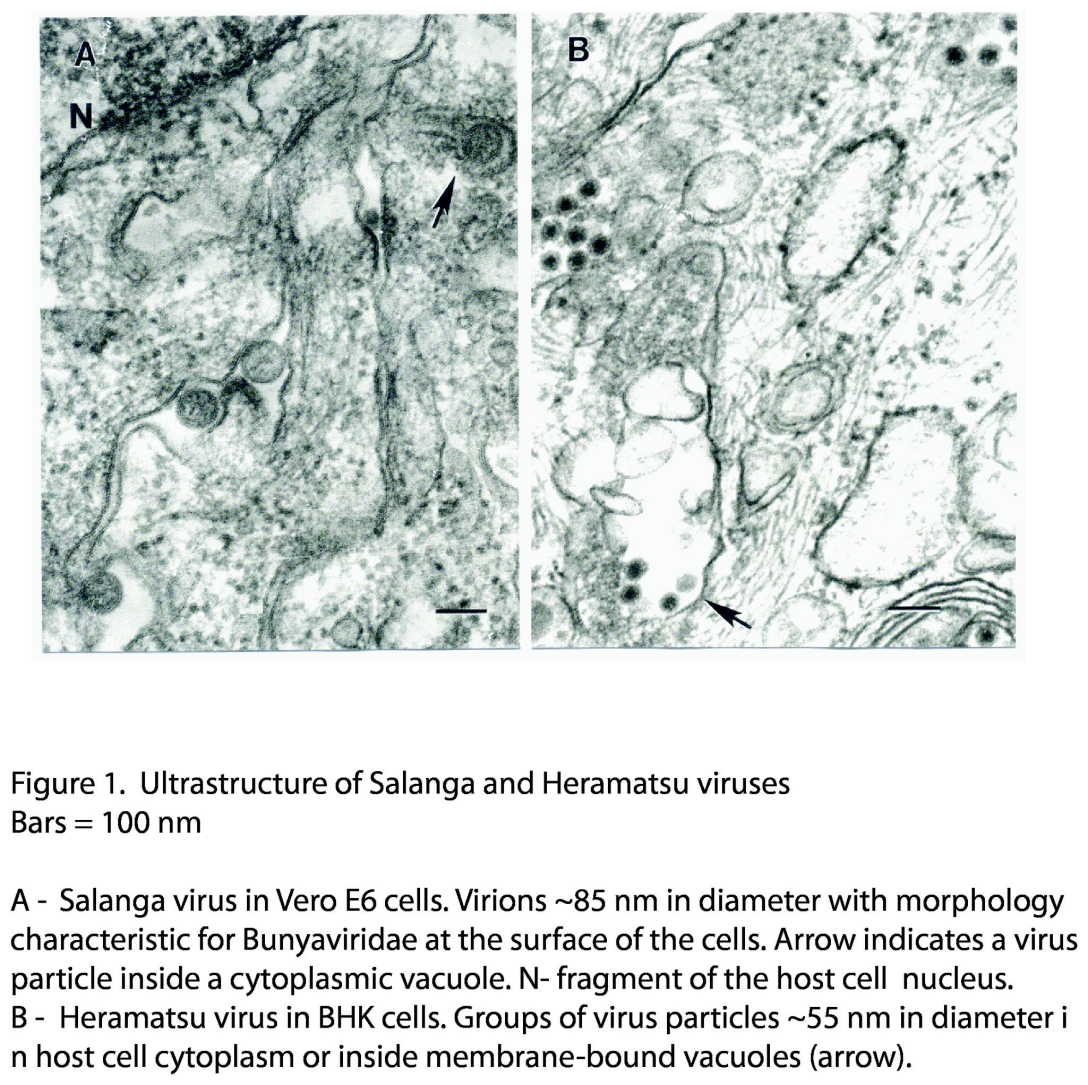
Ultrastructure of Salanga and Heramatsu viruses.

In ultrathin sections of Vero E6 cells infected with Salanga virus, particles ~85 nm in diameter were found either inside cytoplasmic vacuoles containing usually one particle each, or at the cell surface (Figure 1A). They had morphology typical for *Bunyaviridae*. In BHK (baby hamster kidney) cells infected with Heramatsu, virus groups of particles ~55 nm in diameter with dense core typical for *Reoviridae* were found either in host cell cytosol or inside membrane-bound vacuoles (Figure 1B). 

### Serologic Tests

Table 1 and Table 2 show the results of complement fixation (CF) and hemagglutination inhibition (HI) tests with Salanga and 6 other selected phlebovirus antibodies and antigens. By CF test, Salanga was distinct from the other 6 viruses; but by HI test the Salanga antibody reacted with each of the other phlebovirus antigens. Although we were unable to produce a hemagglutinin with the Salanga mouse brain antigen to determine the homologous antibody titer, the results nonetheless indicate that Salanga is a phlebovirus and probably a member of Sandfly fever group. 

**Table 1:** Results of complement fixation test with Salanga and other selected phleboviruses.

Antigen	Complement Fixation test^*^						
	Antibody						
	Naples	Tehran	Karimabad	Toscana	Gordil	St.Floris	Salanga
Naples	256/32	256/32	0	256/32	0	0	0
Tehran	64/128	256/128	0	128/32	0	0	0
Karimabad	32/8	128/8	32/128	32/8	0	0	0
Toscana	64/128	256/128	0	256/128	0	0	0
Gordil	0	16/8	0	32/8	512/≥128	32/≥128	0
St.Floris	0	0	0	8/8	16/≥128	512/≥128	0
Salanga	0	0	0	0	0	0	1024/≥64

* CF titers are reported as the reciprocal of highest antibody dilution/reciprocal of highest antigen dilution.

**Table 2:** Results of hemagglutination inhibition test with Salanga and other selected phleboviruses.

Antibody	Hemagglutination Inhibition test^*^					
	Antigen (4 u.)					
	Naples	Tehran	Karimabad	Toscana	Gordil	St.Floris
Naples	1:320	1:160	1:40	1:80	1:40	1:40
Tehran	1:2560	1:2560	1:160	1:320	1:320	1:320
Karimabad	1:40	1:20	1:320	1:40	0	0
Toscana	1:640	1:640	1:160	1:5120	1:320	1:320
Gordil	1:640	1:640	1:40	1:640	1:5120	1:640
St.Floris	1:640	1:320	0	1:320	1:640	1:2560
Salanga	1:640	1:160	1:320	1:320	1:20	1:40

* HI titers are reported as highest positive antibody dilution.

### Next Generation Sequencing and Sequence Analysis Using VirusHunter Identified Novel Viruses

DNA and RNA were extracted separately from Vero cells that had been inoculated with Salanga virus infected newborn mouse brain homogenate. Total nucleic acid was extracted from Heramatsu virus infected BHK cell culture supernatant and cell lysate independently. Each sample was subjected to reverse-transcription to enable subsequent detection of both RNA and DNA viruses. Following random PCR amplification, the samples were pooled (with barcodes) and then sequenced on the 454 FLX Titanium platform. 20,893 and 8,042 total reads were obtained from Salanga virus DNA and RNA sample preparation respectively. The cell lysate and fluid of Heramatsu virus cell culture were sequenced independently yielding 19,379 and 30,116 reads respectively. 

**Figure 2:**
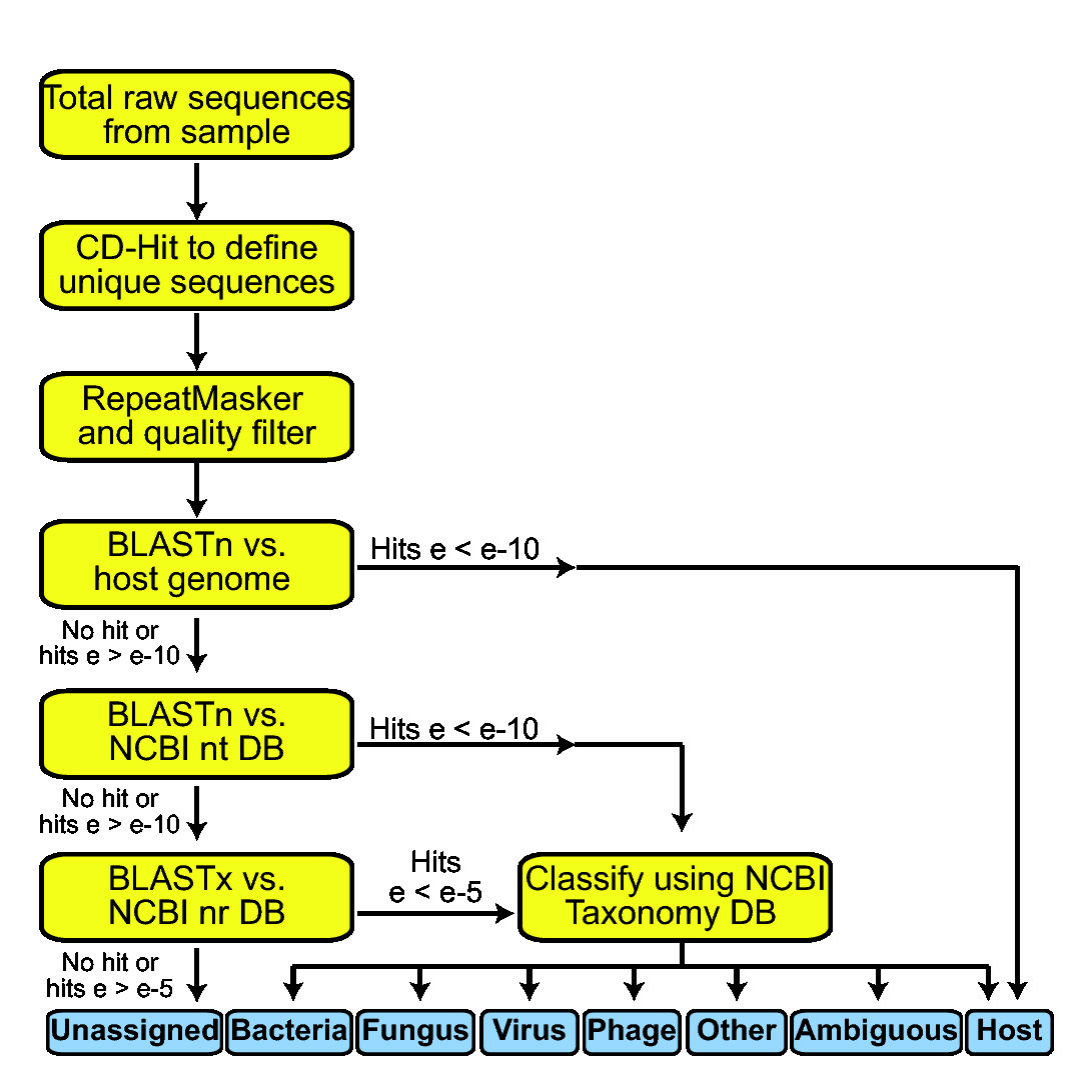
Workflow of VirusHunter.

We used a customized data analysis pipeline, VirusHunter, to identify viral sequences from the raw sequences of each sample. VirusHunter classifies each sequence based on the best BLAST similarity to sequences in reference databases (Figure 2, details see materials and methods). A detailed summary of the results of each step of VirusHunter is available in Files S1, S2 and S3. To give one specific illustration, for the Salanga virus RNA, 8042 total reads were collapsed by CD-HIT [19] (parameters 95% identity over 95% of the length) to 2924 unique sequences. Following repeatmasker analysis 861 reads were removed and 2063 “good sequences” were retained. 1697 of these reads mapped to mammalian genomes. Following the serial BLAST analyses, 144 unique viral sequences that shared 28.6-88.9% sequence identity to viruses in the genus *Phlebovirus* of the family *Bunyaviridae* were identified. No viral sequence was identified from the DNA sample of Salanga virus. From sequencing of total nucleic acid extracted from the cell lysate and fluid of Heramatsu virus cultured in BHK cells, VirusHunter identified 234 and 186 unique viral reads that shared 28.8-86.1% sequence identity to viruses of the *Orbivirus* genus in the family *Reoviridae*. 

### Salanga Virus – Background, Genome Assembly, Sequence Confirmation and Analysis

#### *Bunyaviridae* Background

The family *Bunyaviridae* includes five genera of enveloped, negative-stranded RNA viruses: *Orthobunyavirus*, *Hantavirus*, *Nairovirus*, *Phlebovirus* and *Tospovirus*. Except tospoviruses which infect plants, the other viruses in the family infect vertebrates, and they include multiple causative agents of haemorrhagic fever such as Crimean-Congo hemorrhagic fever virus (CCHFV), Ngari virus (NRIV), Hantaan (HTNV) and Rift Valley fever virus (RVFV). Most viruses in the *Bunyaviridae* family are carried and transmitted by arthropods (e.g., mosquitoes, ticks, and other insects). Many bunyaviruses are classified as emerging pathogens due to their recent increased incidence in new vertebrate hosts and in new geographical locations. Bunyavirus genomes contain three negative-sense, single-stranded RNA segments, designated as large (L), medium (M), and small (S) segment. 

#### Salanga Virus Genome Assembly

We used all individual reads with detectable similarity to bunyaviruses as well as all sequences that did not share detectable sequence similarity with any sequence in Genbank databases as inputs for assembly using the Newbler assembler. Four contigs of length 555-3853 bp were identified. These contigs were most closely related to Durania virus, Ariquemes virus, Leticia virus (Phlebovirus sp. Co Ar 171616) and Sandfly fever sicilian virus with 61.0, 44.3, 51.7 and 26.3% aa identity respectively. Of the 4 contigs, 2 mapped to the L segment, 1 mapped to the M segment and 1 mapped to the S segment.

#### Salanga Virus Genome Confirmation

Using RT-PCR amplification followed by Sanger sequencing we closed the gap between the two initial L segment contigs. Additional RACE (Rapid Amplification of cDNA Ends) reactions extended the initial contigs and yielded final contigs of length 6513 bp (L segment), 4412 bp (M segment) and 1795 bp (S segment). Over-lapping RT-PCR amplification and Sanger sequencing were employed to confirm the sequences with at least 2 fold coverage for L segment nucleotide 174-6225, M segment nucleotide 280 - 4312 and the S segment nucleotide 51-1675. Using standard gene prediction tools, open reading frames were predicted in each segment. No further effort was made to completely sequence the 5’ or 3’ termini of the viral genome segments. 

### Salanga Virus Genome Segment Description

We obtained 6513 bp of the Salanga virus L segment (Table 3). A single open reading frame of 2123 aa was predicted. The predicted ORF was most closely related to the polymerase of Arbia virus (NR database dated February 11, 2012), a Salehabad species group virus in the *Phlebovirus* genus [20], with 59% aa identity (e value of 0.0E+00). This is consistent with the genome structure of all other bunyaviruses in which the L segments encode a single protein -- the viral RNA-dependent RNA polymerase (RdRp). The RdRp is a multifunctional protein that is responsible for the generation of RNA-replication and mRNA-transcription products from their respective viral templates. 

**Table 3:** Salanga virus genomic segments.

Segment	Length (bp)	Encoded protein	Protein (aa)	homolog	length (aa)	aligned region (aa)	identity (aa%)	significance
L	6513	Pol	2123*	Polymerase [Arbia virus]	2096	1263/2131	59	0.0E+00
M	4412	G1, G2, NSm	1398*	Polyprotein [Arbia virus]	1331	553/1390	40	0.0E+00
S	1795	NSs	269*	Non-structural protein [Durania virus]	276	54/211	26	7.00E-15
		N	245*	Nucleocapsid [Phlebovirus CoAr 171616]	244	123/238	52	1.00E-89

* Full length open reading frame from start codon to stop codon.

**Figure 3:**
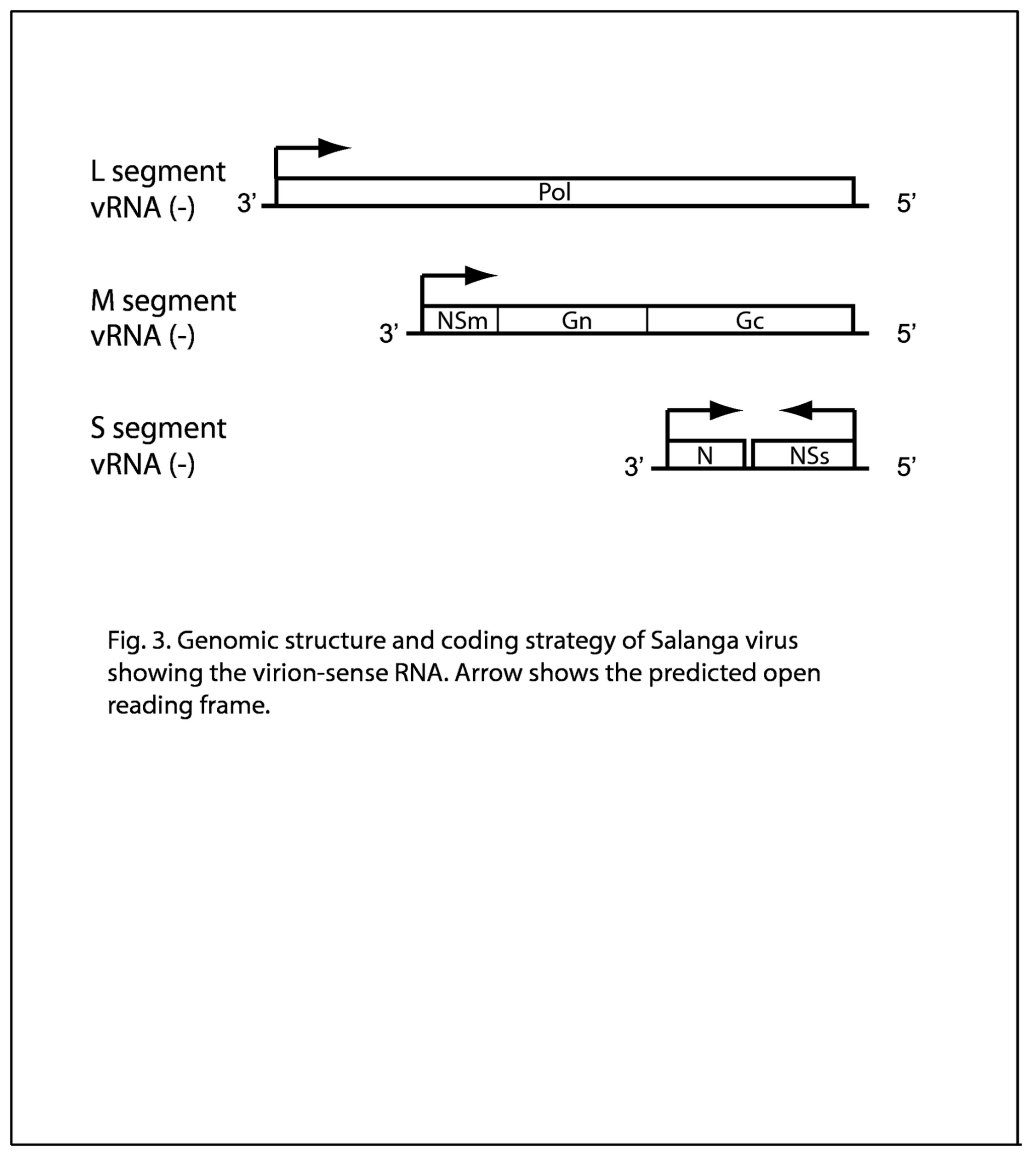
Genomic structure and coding strategy of Salanga virus. Predicted open reading frames indicated by boxes with arrows indicating the directionality of the open reading frame.

We obtained 4412 bp of the Salanga virus M segment and a single open reading frame of 1398 aa was predicted. The predicted ORF was most closely related to the polyprotein of Arbia virus (NR database dated February 11, 2012), with 40% aa identity (e value of 0.0E+00). The M segment of all bunyaviruses encodes the glycoprotein precursor which is subsequently processed to yield two glycoproteins, Gn and Gc. However, the M segments of orthobunyaviruses, phleboviruses and tospoviruses encode an additional protein, the nonstructural protein NSm. The NSm protein is an integral membrane protein localized in the Golgi complex and it is important for virus assembly and morphogenesis [21]. Interestingly, the location of the NSm coding region differs by genera. The NSm protein of orthobunyaviruses is encoded between the Gn and Gc proteins whereas the NSm protein of phleboviruses is N-terminal of both Gn and Gc proteins (at the 3' end of the negative sense genomic RNA). The Salanga virus M segment was predicted to encode a NSm protein at the 3' end of the viral RNA, consistent with the location observed in phleboviruses (Figure 3). 

We obtained 1795 bp of the Salanga virus S segment. Two open reading frames of 269 and 245 aa were predicted. One ORF was predicted on the negative sense genomic strand while the other was encoded on the anti-genomic strand. The predicted ORF positioned at the 5’ end of the genomic strand was most closely related to the nonstructural protein (NSs) encoded in the S segment of Durania virus, an Aguacate group phlebovirus [22], with 26% aa identity (e value of 7.0E-15, NR database dated Nov. 12, 2012). The predicted ORF at the 3’ end of the genomic strand was most closely related to the nucleocapsid (N) protein of Leticia virus, a Punta Toro group phlebovirus, with 52% aa identify (e value of 1.0E-89, NR database dated Nov. 12, 2012). The S segments of all bunyaviruses encode the protein N whose primary role is to encapsidate the viral RNA-replication products to form the ribonucleoprotein complex. The protein N is always encoded in the anti-genomic strand. The S segments of orthobunyavirus, phlebovirus and tospovirus also encode a nonstructural protein called NSs whose primary role is modulating the host-cell antiviral response through diverse innate-immunity pathways. NSs is encoded in the anti-genomic strand in orthobunyaviruses but in the genomic strand in phleboviruses and tospoviruses. These coding strategies are generally conserved and aid in bunyavirus classification. This is consistent with its classification as a phlebovirus. 

#### Salanga Virus Phylogenetic Analysis

We performed phylogenetic analysis using the RNA-dependent RNA polymerase protein sequence of Salanga virus encoded on the L Segment and representative members of the *Phlebovirus* genus within the *Bunyaviridae* family. Both Neighbor-joining and maximum parsimony trees (Figure 3) of the amino acid sequence yielded the same tree topology and demonstrated that Salanga virus was most closely related to, but highly divergent from, the viruses in the *Phlebovirus* genus. Therefore Salanga virus should be classified as a new species within the *Phlebovirus* genus in the *Bunyaviridae* family.

### Heramatsu Orbivirus – Background, Genome Assembly, Sequence Confirmation and Analysis

#### Orbivirus Background

The family *Reoviridae* contains 15 genera that differ substantially in genetic, structural and biological properties. The *Orbivirus* genus contains 22 serogroups (species) and at least 160 different serotypes (strains) [23]. The orbiviruses have genomes consisting of 10 distinct segments of linear double-stranded RNA (dsRNA) that encode seven structural and at least three non-structural proteins. Bluetongue virus (BTV), the type species of the genus has 26 recognized serotypes, and African horse sickness virus (AHSV) which has 9 serotypes are the most economically important members of this genus. Orbiviruses are transmitted to animals primarily by arthropod vectors, including Culicoides midges, mosquitoes, black flies, sandflies, or ticks. Arthropod-transmitted viruses are important causes of disease in humans and animals, and it has been proposed that climate change will alter the distribution and severity of BTV infection and of other arboviral diseases [24].

#### Heramatsu Orbivirus Genome Assembly

To obtain the best genome assembly, all sequences with detectable similarity to orbiviruses as well as all sequences that did not share detectable similarity with any sequence in the databases were pooled together and assembled using Newbler assembler. Assembled contigs were compared with NCBI nr database (downloaded on January 25^th^, 2012) using BLASTx to identify all viral contigs. Fourteen putative viral contigs with length 131-3835 bp were identified that were most closely related to orbiviruses. The contigs shared 26.7- 71.2 % aa identity to Chuzan virus, AHSV, or BTV, each of which belongs to the *Orbivirus* genus of the *Reoviridae*. Chuzan, also known as Kasba virus, is a strain (serotype) of Palyam virus.

#### Heramatsu Orbivirus Genome Segment Description

The 14 contigs generated from Heramatsu virus were mapped to reference orbivirus genome segments using BLAST and annotated. 13 of the 14 Heramatsu virus contigs showed significant sequence similarity to BTV genomic segments. One of the contigs did not have any detectable similarity to any BTV sequence; rather it possessed highest sequence similarity to the segment 2 of Chuzan virus with 27% aa identity over a 187 aa stretch (e value of 6E-07). Segment 2 of Chuzan virus encodes the outer capsid protein VP2. Reassortment of genome segments can contribute to virus diversity and is known to occur in orbiviruses [25]; the distinct relationship of segment 2 may be the result of reassortment. The RNA segment name, encoded protein as well as putative function were assigned according to the nomenclature of the Orbivirus type species BTV (Table 4) following recommended rules [23].

**Table 4:** Heramatsu orbivirus genomic segments and BLAST alignment results.

Segment	Length (bp)	Length (aa)	Top BLAST hit	Length (aa)	Aligned region (aa)	ID (aa%)	Significance	Putative function
S1	3835	1278	African horse sickness virus 1 VP1 (S1)	1305	711/1290	55	0.0E+00	RNA-dependent RNA polymerase
S2	2611	853*	Chuzan virus VP2 (S2)	1002	64/266	24	6.0E-09	Outer capsid protein VP2
S3	2603	867	African horse sickness virus 1 VP3 (S3)	905	495/867	57	0.0E+00	Inner shell protein (T2)
S4	1915	624^#^	Bluetongue virus 2 VP4 (S4)	644	318/630	50	0.0E+00	Capping enzyme (CaP)
S5	1672	539*	Palyam virus NS1 (S5)	545	168/535	31	6.0E-87	Non-structural protein NS1, forms Tubules (TuP)
S6	1526	500^#^	Bluetongue virus 7 VP5 (S6)	526	237/501	47	8.0E-152	Outer capsid protein VP5
S7	956	318	Pata virus VP7 (S7)	348	121/316	38	1.0E-72	Inner shell protein (T13)
S8	1004	320*	Bluetongue virus 8 NS2 (S8)	354	100/349	29	1.0E-43	Non-structural protein NS2, viral inclusion body matrix protein (ViP)
S9	738	240^$^	Palyam virus VP6 (S9)	272	92/258	36	2.0E-30	ssRNA and dsRNA binding helicase (Hel)
S10	683	204*	African horsesickness virus NS3 (S10)	217	79/200	40	1.0E-39	Glycoprotein

* Full length open reading frame from start codon to stop codon.

$ Only have start codon. # Only have stop codon.

#### Heramatsu Orbivirus Genome Sequence Confirmation

Orbivirus genomes consist of 10 RNA segments. For most of the Heramatsu virus segments, single contigs were assembled that were approximately the same size the corresponding segment of the most closely related orbivirus in Genbank. The exceptions to this were segment 3 for which we obtained four contigs of length 869, 428, 260 and 886 bp and segment 9 for which we had two contigs of length 131 and 606 bp. These gaps were closed by PCR followed by Sanger sequencing of the cloned PCR amplicons. For segment 3, a final contig of 2603 nt was generated. Overlapping RT-PCR amplification and Sanger sequencing were employed to sequence the segment 3 contig nucleotide 21-2559 with at least 2-fold coverage. For segment 9 a final contig of 738 bp was obtained, of which nucleotides 8-723 were confirmed with at least 2-fold coverage. While these contigs did not represent the complete sequences of the genome segments, they sufficed for taxonomic classification and thus no effort was made to obtain the terminal sequences of any of the segments. 

#### Heramatsu Orbivirus Phylogenetic Analysis

**Figure 4:**
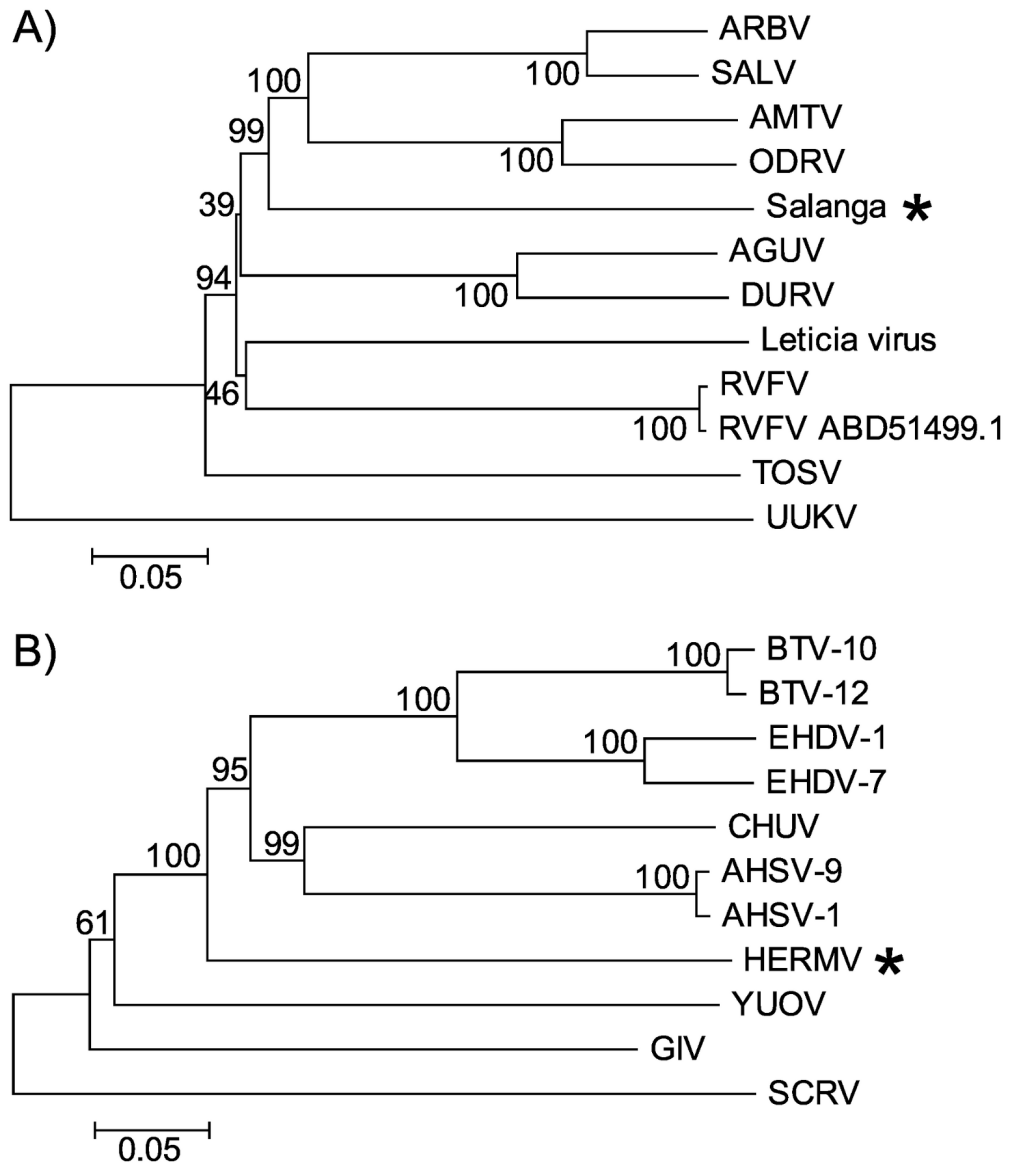
Phylogenetic analysis of Salanga and Heramatsu Orbivirus virus. Phylogenetic tree comparing RNA-dependent RNA polymerase of (A) Salanga virus with representative members of the *Phlebovirus* genus within the *Bunyaviridae* family and (B) Heramatsu Orbivirus with representative members of the *Orbivirus* genus within the *Reoviridae* family. Phylogenetic trees were constructed by using the neighbor-joining method in the MEGA5 program with 1000 bootstrap replicates. The percentage of replicate trees in which the associated taxa clustered together in the bootstrap test is shown next to the branches. The tree is drawn to scale, with branch lengths measured in the number of substitutions per site.

To understand the relationship of Heramatsu virus to other orbiviruses we performed phylogenetic analysis using the RNA-dependent RNA polymerase protein sequence of Heramatsu virus encoded on Segment 1 and representative members of the *Orbivirus* genus within the *Reoviridae* family. Both Neighbor-joining and maximum parsimony trees (Figure 4) of the amino acid sequence yielded the same tree topology and demonstrated that Heramatsu virus is most closely related to, but highly divergent from, the viruses in the *Orbivirus* genus. According to previously published criteria, RNA polymerase sequences of viruses belonging to a single genus within the family *Reoviridae* share >30% amino acid sequence identity [26]. Based on this criterion, Heramatsu virus should be classified as an *Orbivirus*. 

## Conclusion

We utilized a combination of traditional virology methods, such as electron microscopy and serological analysis, and NGS sequencing with subsequent bioinformatics analysis using VirusHunter, to identify and taxonomically classify two previously isolated, but poorly characterized, viruses from the WRCEVA collection. Sequence analysis demonstrated that the viruses were highly divergent from previously described viruses. Salanga virus was classified as a novel member of the *Phlebovirus* genus in the *Bunyaviridae* while Heramatsu virus was determined to be a novel member of the *Orbivirus* genus in the *Reoviridae*. Both the *Orbivirus* and *Phlebovirus* genera include many economic important viruses or serious human pathogens. The sequencing and classification of these two viruses will be a valuable addition to studies of arboviruses and emerging viruses. 

## Materials and Methods

### Virus Strain

Salanga virus (strain AnB 904a) was originally isolated from a rodent (*Aethomys medicatus*) collected in September 1971 at Salanga, Central African Republic. The Heramatsu virus (strain KY-663) used in this study was originally isolated from the blood of a bat (*Myotis macrodactylus*) collected in a mine at Heramatsu, Kagoshima, Japan in July 18^th^, 1965. 

### Transmission Electron Microscopy

For ultrastructural analysis in ultrathin sections, infected Vero or BHK cells were fixed for at least 1 h in a mixture of 2.5% formaldehyde prepared from paraformaldehyde powder, and 0.1% glutaraldehyde in 0.05 M cacodylate buffer pH 7.3 to which 0.03% picric acid and 0.03% CaCl_2_ were added. The monolayers were washed in 0.1 M cacodylate buffer, cells were scraped off and processed further as a pellet. The pellets were post-fixed in 1% OsO_4_ in 0.1 M cacodylate buffer pH 7.3 for 1 h, washed with distilled water and en bloc stained with 2% aqueous uranyl acetate for 20 min at 60°C. The pellets were dehydrated in ethanol, processed through propylene oxide and embedded in Poly/Bed 812 (Polysciences, Warrington, PA). Ultrathin sections were cut on Leica EM UC7 ultramicrotome (Leica Microsystems, Buffalo Grove, IL), stained with lead citrate and examined in a Philips 201 transmission electron microscope at 60 kV. 

### Antigens and Immune Reagents

Antigens used in serologic tests were infected newborn mouse brains prepared by the sucrose/acetone extraction method [27]. Specific hyperimmune mouse ascitic fluids were prepared at the WRCEVA against each of the viruses used in this study. The immunization schedule consisted of four intraperitoneal injections given at weekly intervals. Immunogens were 10% suspensions of homogenized infected mouse brain in PBS, mixed with Freund’s adjuvant just prior to inoculation. Sarcoma 180 cells were also given intraperitoneally with the final immunization in order to induce ascites formation. All animal experiments were carried out under an animal protocol approved by the University of Texas Medical Branch IACUC committee. 

### Serologic Tests

Hemagglutination inhibition (HI) tests were done in microtiter plates, as previously described [28], using four units of antigen. The HI titer was read after overnight incubation of antigen and antibody at 4°C. Complement-fixation (CF) tests were performed by the microtiter technique [28], using two units of guinea pig complement and overnight incubation of the antigen and antibody at 4°C. The CF titers were recorded as the highest dilutions giving 3+ or 4+ fixation of complement on a scale of 0 to 4+. 

### Preparation and Sequencing of Viral DNA and RNA

Total nucleic acid was extracted from Heramatsu virus infected BHK cell culture supernatant and cell lysate independently with a Qiagen DNeasy kit according to the manufacturer’s instructions. DNA and RNA were extracted from Salanga virus infected culture of Vero E6 cells cell separately. Both cell lines were obtained from the American Type Culture Collection (Manassas, VA). 

Nucleic acid from each sample was reverse-transcribed to enable subsequent detection of both RNA and DNA viruses and was amplified as previously described [29,30]. Amplification products were pooled, adaptor-ligated and sequenced at the Washington University Genome Sequencing Center on the 454 GS-FLX platform (454 Life Sciences). Sequences were then trimmed to remove primer B sequences prior to assembly using the Newbler program (454 Life Sciences, Branford, CT). 

### Next Generation Sequencing Data Analysis

Because the nucleic acid used for sequencing contained a mixture of host cell DNA and virus RNA, we used a customized informatics pipeline VirusHunter to identify viral sequences. The VirusHunter pipeline is controlled by a master Perl script VirusHunter.pl, which executes each step in the pipeline. The input to the pipeline is a directory path. The directory holds sequencing data from different samples. Each sample has its own directory with a single file containing FASTA format sequence reads. 

The workflow of the pipeline is shown in Figure 2. Below is a brief description of each step.

1 Remove redundant sequences. Identical or nearly-identical sequences are frequently present in NGS data, either due to the sheer depth of NGS or because many of the pre-sequencing sample preparation methods involve PCR amplification. To reduce the computing cost of downstream analysis, CD-HIT [19] is first used to cluster similar sequences. The default parameters in VirusHunter are set to cluster sequences that share ≥ 95% identity over 95% of the sequence length. The longest sequence from each cluster is retained as the representative sequence and used for downstream analysis. These are the “unique sequences”.
2 Mask repetitive sequences and sequence quality control. Many eukaryotic genomes contain stretches of highly repetitive DNA sequences which cause problems in BLAST-based similarity searches and result in high rates of false-positive alignments. RepeatMasker (http://www.repeatmasker.org) is used to mask interspersed repeats and low complexity DNA sequences. A sequence fails the quality control criteria if it does not contain a stretch of at least 50 consecutive non-“N” nucleotides (i.e., “Filtered sequence”) or if greater than 40% of the total length of the sequence is masked (i.e., "low complexity sequence"). These sequences are removed from further analysis. Remaining sequences are “good sequences”.
3 Filtering host sequences. Sequences are subjected to BLASTn [31] alignment against the appropriate host genome (default Human). BLASTn output files are parsed using BioPerl [32]. Any sequence that shares significant similarity (e value ≤ 1E-10) is classified as "Host" and removed from further analysis. Any desired sequence database can be used for filtering. For Salanga virus, which was a unique case wherein the virus was cultured in Vero (African Green Monkey) cells, but inoculated with infected newborn mouse brain homogenate, we used the reference Human genome from the 1000 Genomes Project as reference (ftp://ftp.ncbi.nlm.nih.gov/1000genomes/ftp/technical/reference/) for simplicity; for Heramatsu virus, which was cultured in the BHK (hamster) cell line we used the golden hamster *Mesocricetus auratus* genome as the reference (GenBank Assembly ID: GCA_000349665.1).
4 BLASTn against NCBI nt database. Sequences retained from the previous step are queried against the NCBI nt database using BLASTn. Sequences with significant hits (e value cutoff 1E-10) are broadly classified as human, mouse, fungal, bacterial, phage, viral or other based on the taxonomy identity of the best BLAST hit. The NCBI gi – taxid data for nucleotide sequences are uploaded to a MySQL database. The gi number of the best BLAST hit is used to query the database to obtain the taxonomy ID which is in turn used to retrieve the full taxonomy lineage using BioPerl. In some instances, one query has two or more hits with the same e value. If a sequence aligns to both a virus and a sequence derived from another organism type (e.g. bacteria or fungi) with the same e value it is classified as “ambiguous”. All eukaryotic viral sequences are further classified into viral families based on the taxonomy ID of the best hit. Sequences without hits progress to the next step.
5 BLASTx against NCBI nr database. Sequences retained from the previous step are queried against the NCBI nr database using BLASTx (e-value cutoff 1E-5). BLASTx output files are parsed and sequences are phylotyped as described in the previous step. Sequences without any significant hit are placed in the “unassigned” category.
6 Report of the Findings

The final output of the VirusHunter pipeline is a single file summarizing all the viruses identified in each dataset in the input directory. The pipeline can be customized to generate similar outputs for other bacterial, fungal or parasitic sequences. 

VirusHunter is written in Perl. It uses shell scripting, the BioPerl library, MySQL database, CD-HIT, RepeatMasker and NCBI BLAST suite. The pipeline is fully automated and high-throughput. All components are organized in a hierarchical set of readily modifiable scripts, and multiple copies of the pipeline can be run in parallel. The pipeline is designed for running on a high performance computing cluster using GridEngine as the job scheduler. It can be easily modified to use other job management software. The pipeline can be easily customized, for example to replace the human genome database with that of a different host. Installation and configuration of VirusHunter requires basic knowledge of Perl, MySQL database and Linux system administration. Distribution and source code for VirusHunter 1.0 are available at (http://www.ibridgenetwork.org/wustl/virushunter). 

### Assembly of the Viral Genome and Genome Annotation

Sequences identified as viral as well as sequences that had no significant hit to any sequence in the NR and NT databases were assembled using Newbler (454 Life Sciences, Branford, CT) with default parameters. ORFs were predicted and annotated using Artemis [33]. Multiple sequence alignments were performed with ClustalW [34]. Phylogenetic analysis was performed using both neighbor-joining method and maximum parsimony method in MEGA5 program [35] with 1000 bootstrap replicates. Trees with the same topology were generated using both methods for all the data sets used in the study. Phylogenetic trees were visualized using TreeView [36]. 

#### Viruses Analyzed and Sequence Accession Numbers Used for Analyses

The viruses analyzed included the following genera, species, and strains: 

#### *Bunyaviridae Phlebovirus* Genus

Aguacate virus (AGUV, YP_004414703.1), Arbia virus (ARBV, AGA82737.1), Arumowot virus (AMTV, AEF30501.1), Durania virus (DURV, AEB70976.1), Leticia virus (AEL29649.1, also named Phlebovirus CoAr 171616), Odrenisrou virus (ODRV, AEL29670.1), Rift Valley fever virus (RVFV, YP_003848704.1), Rift Valley fever virus (RVFV_ABD51499.1, ABD51499.1), Salehabad virus (SALV, AGA82741.1), Toscana virus (TOSV, CAA48478.1), Uukuniemi virus (UUKV, BAA01590.1).

#### *Reoviridae Orbivirus* Genus

African horsesickness virus serotype 1 (AHSV-1, CAP04840.1), African horsesickness virus serotype 9 (AHSV-9, AAC40586.1), Bluetongue virus Serotype 10 (BTV-10, YP_052968.1), Bluetongue virus Serotype 12 (BTV-12, AGJ83521.1), Chuzan virus (CHUV, BAA76549.1), Epizootic hemorrhagic disease virus serotype 1 / strain New Jersey (EHDV-1, YP_003240108.1), Epizootic hemorrhagic disease virus serotype 7 / strain CSIRO 775 (EHDV-7, CAN99553.1), Great Island virus (GIV, YP_003896058.1). St Croix River virus (SCRV, YP_052942.1), Yunnan orbivirus (YUOV, YP_443925.1). 

#### Nucleotide Sequence Accession Number

The sequence of the Salanga virus and Heramatsu Orbivirus (HERMV) KY-663 genomic fragments has been deposited in the NCBI database under GenBank accession number KC669549 - KC669551 and KC669539 - KC669548 respectively. Raw data is deposited in the Sequence Read Archive (SRA) under accession number SRR959764.

## Supporting Information

File S1 **VirusHunter summary output file of the Salanga virus RNA sequencing data.**
(TXT)

File S2 **VirusHunter summary output file of the Heramatsu Orbivirus (HERMV) KY-663 cell culture lysate sequencing data.**
(TXT)

File S3 **VirusHunter summary output file of the Heramatsu Orbivirus (HERMV) KY-663 cell culture fluid sequencing data.**
(TXT)
